# Integrated Physiological and Transcriptome Analyses of Wild Jujube (*Ziziphus jujuba* var. *spinosa*) Under Drought Stress

**DOI:** 10.3390/ijms27062669

**Published:** 2026-03-14

**Authors:** Quangang Liu, Jie Lu, Yuejia Liu, Rui Liang, Jianhua Chen, Qingbai Liu, Shengjun Dong

**Affiliations:** 1 College of Forestry, Shenyang Agricultural University, Shenyang 110866, China; liuquangang007@126.com (Q.L.); 15542494533@163.com (J.L.); 2024240978@stu.syau.edu.cn (Y.L.); 2025241012@stu.syau.edu.cn (R.L.); 2023500049@syau.edu.cn (J.C.); 2 Key Laboratory for Silviculture of Liaoning Province, Shenyang Agricultural University, Shenyang 110866, China

**Keywords:** transcriptome, drought stress, wild jujube (*Ziziphus jujuba* var. *spinosa*), gene expression, drought-resistant

## Abstract

Drought stress is a significant environmental factor affecting plant growth, fruit quality and distribution. Wild jujube is an important species of eco-economic forest tree. In this study, two wild jujube families, ‘NO. 1’ (tolerant) and ‘NO. 5’ (sensitive), which show significant differences in morphological and physiological indicators in drought treatment, are considered. Compared with the ‘NO. 5’, the ‘NO. 1’ exhibited lower water loss, leaf yellowing and abscission rates, as well as reduced malondialdehyde (MDA) content, while showing higher superoxide dismutase (SOD) activity and elevated levels of soluble sugars (SS), soluble proteins (SP), and proline (Pro). In contrast, the ‘NO. 5’ suffered more severe damage to leaf epidermal cells compared with the ‘NO. 1’, accompanied by a significant decline in net photosynthetic rate (*A*) and instantaneous water use efficiency (WUEi). Transcriptomic profiles between two wild jujube families with markedly different drought responses (withholding water for 15 days) are shown. The two wild jujube families included 3238 up-regulated and 2675 down-regulated differentially expressed genes (DEGs). Many DEGs enriched in the GO and KEGG pathways are related to antioxidant activity, transmembrane transport, carbohydrate biosynthesis and metabolism, plant hormones, and photosynthesis. The biosynthesis of amino acids, the MAPK signaling pathway, plant hormone signal transduction, and flavonoid and alkaloid biosynthesis were the transcriptome modifications most significantly altered by drought stress. Real-time quantitative polymerase chain reaction (RT-qPCR) was used to verify the precision of the RNA-seq data. *ZjJIP23-1*, *ZjbZIP53*, *ZjSPS8*, *ZjCAO*, *ZjADH1* and *ZjERF39* may play important roles in the drought tolerance of the wild jujube. This study provides a solid foundation for further studies on the complex mechanisms and breeding of drought-resistant plants in wild jujube.

## 1. Introduction

Wild jujube (*Ziziphus jujuba* Mill. var. *spinosa* (Bunge) Hu ex H. F. Chou) is native to China and is recognized as the ancestor of common jujube (*Ziziphus jujuba*) [1]. As a perennial shrub or small tree of the Rhamnaceae family, it is now extensively cultivated across Asia, Europe, and the Americas [2]. It is termed sour jujube exactly due to the sweet and sour flavor of its fruits. This plant boasts considerable edible, medicinal, and healthcare values [3]. Its fruits are applicable for winemaking and juice extraction; its flowers serve as a high-quality nectar source; its core shells can be processed into activated carbon; its leaves also exhibit sedative and hypnotic properties [3]. The mature seeds of wild jujube, commonly referred to as suanzaoren, constitute a traditional Chinese medicinal herb first documented in Shennong’s Classic of Materia Medica. They exert significant effects in treating various diseases, are often utilized to alleviate insomnia, anxiety, and depression, and can be processed into functional foods to improve sleep quality, enhance learning and memory capabilities, and strengthen immunity [4]. Furthermore, wild jujube demonstrates extremely strong stress resistance to adverse environmental conditions, playing a crucial role in windbreak and sand fixation, soil and water conservation, and ecological restoration, and making it an ideal tree species for afforesting barren hills [5]. Thanks to its remarkable tolerance to drought and poor soils, wild jujube is frequently employed as rootstock for grafting common jujube trees [6].

Drought is one of the primary environmental factors impacting plant growth, development, and distribution, exerting substantial detrimental effects [7]. Approximately one-third of the Earth’s land area is composed of arid and semi-arid regions. Under drought stress, plants undergo a series of physiological alterations to adapt to and resist this adverse condition [8]. Plants achieve osmotic regulation via the accumulation of metabolites, with soluble sugars (SS), soluble proteins (SP), and proline (Pro) forming the core osmotic protection system [9]. These substances sustain cellular turgor and osmotic homeostasis, guaranteeing the normal operation of physiological activities. SS enhances cellular dehydration tolerance by regulating water potential balance, while SP remarkably improves tissue water retention capacity due to its strong hydrophilicity [10]. As a key osmotic buffer, elevated Pro concentration effectively reduces water loss and stabilizes the conformation of biological macromolecules [11]. On the other hand, malondialdehyde (MDA), a membrane lipid peroxidation product, reflects the degree of cellular membrane peroxidation [12]. As an important marker of cellular damage, its concentration directly indicates a plant’s drought resistance. Drought-induced rapid accumulation of reactive oxygen species (ROS) disrupts redox homeostasis, which in turn impairs the membrane system [13]. In this process, superoxide dismutase (SOD), peroxidase (POD), and catalase (CAT) form the core of the antioxidant defense system [7]. SOD alleviates oxidative damage by scavenging O^2−^ and regulating the concentrations of O^2−^ and H_2_O_2_. POD not only decomposes H_2_O_2_ but is also closely related to cellular metabolic activities [14]. When cells perceive drought signals, H_2_O_2_ acts as a secondary messenger to activate protective mechanisms, yet its excessive accumulation can trigger programmed cell death (PCD) [15]. As a specific H_2_O_2_-scavenging enzyme, CAT converts H_2_O_2_ into H_2_O and O_2_, playing a pivotal role in the antioxidant cascade [16].

In recent years, as global climate change intensifies, the adverse impacts of drought on plant growth and yield have grown increasingly notable, and investigating the molecular mechanisms governing plant drought resistance has attracted extensive attention [17]. Transcription factors (TFs) are capable of binding to *cis*-acting elements within the promoters of stress-responsive genes and have been shown to serve critical functions in enhancing drought stress tolerance [18]. At present, in model plants like *Arabidopsis* and other major economic crops, genetic engineering techniques have been applied to screen and verify the pivotal roles of transcription factor families, such as AP2/ERF, WRKY, MYB, NAC and bZIP in coping with drought stress [19]. By utilizing key drought-responsive genes and their interaction relationships, a preliminary molecular regulatory network made up of multiple structural genes and TFs has been established [20,21]. *MhERF113-like*, a drought-induced and leaf-preferential gene from *Malus hupehensis*, positively regulates drought tolerance in both apple and tomato plants by reducing oxidative damage, and activating stress-related (*SlDREB* and *SlRD29*) antioxidant genes (*SlAPX1* and *SlSOD*) [22]. *VvWRKY70*, rapidly induced by drought in grapevine, enhances drought tolerance by regulating stomatal aperture, osmotic adjustment, antioxidant systems, and the expression of drought-responsive (*VvRD22*, *VvRD29A* and *VvDREB2A*) and defense-related genes (*VvPOD4* and *VvP5CS*) [23]. *CaADBZ1*, a pepper A-group bZIP transcription factor induced by dehydration and ABA, enhances plant dehydration tolerance by positively regulating ABA sensitivity and the expression of dehydration-responsive genes, including *CaOSR1* [24]. However, the research progress in elucidating the molecular mechanisms of drought stress responses in woody plants has been relatively slow, attributed to their prolonged growth cycle, low genetic transformation efficiency, and intricate genetic regulation of traits. Consequently, the genomic and genetic architecture underlying drought stress responses in forest trees remains unclear, and the molecular mechanisms of key regulatory genes need to be further explored. Currently, the integration of physiological indices and transcriptomic data has laid a solid foundation for unraveling the associated molecular mechanisms [7]. Several RNA-seq studies have identified drought resistance-related genes in plants, which encode proteins involved in hormone signal transduction, osmolyte accumulation, membrane permeability regulation, ion transport, and redox reactions [25,26,27].

Wild jujube serves as a crucial eco-economic forest species. To comprehensively elucidate the key genes and associated pathways involved in drought stress responses, two *Z. jujuba* var. *spinosa* families with contrasting drought resistance (drought-tolerant ‘NO. 1’ and drought-sensitive ‘NO. 5’) were investigated from phenotypic, anatomical, and physiological perspectives. RNA-seq was adopted to analyze transcriptional changes in the two families under drought stress, which helped identify potential molecular regulatory pathways of drought responses and predict candidate genes related to drought resistance in *Z. jujuba* var. *spinosa*. This study facilitates the elucidation of adaptation mechanisms of wild jujube seedlings to drought stress, spanning from macroscopic to microscopic levels. It lays a foundation for subsequent early identification of drought-resistant germplasm resources, functional verification, and clarification of molecular mechanisms. Meanwhile, it provides up-to-date research insights and genetic resources for enhancing the drought resistance of wild jujube.

## 2. Results

### 2.1. Morphological and Physiological Responses of Wild Jujube Under Drought Stress

**Figure 1:**
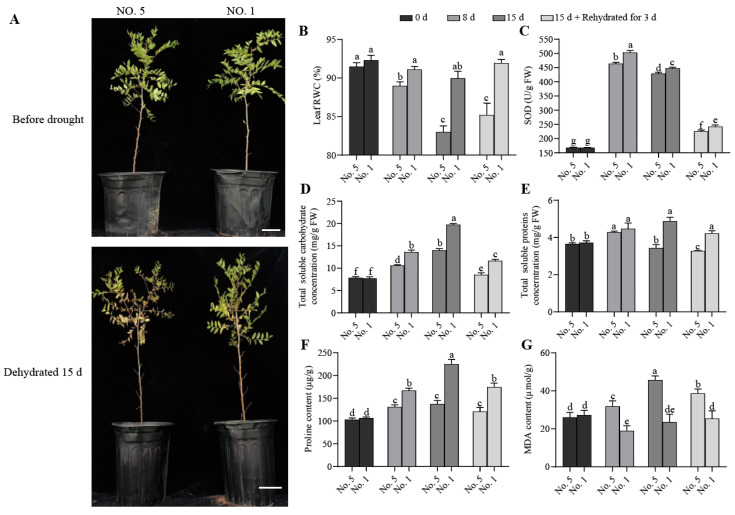
Drought stress treatment and physiological indicators were determined in leaves of *Ziziphus jujuba* var. *spinosa* from two families ‘NO. 1’ and ‘NO. 5’. (**A**) Phenotypic observations under 15 days of drought stress. Scale bar: 5 cm, (**B**) leaf relative water content (RWC), (**C**) SOD activity, (**D**) soluble carbohydrate content, (**E**) soluble protein content, (**F**) Proline content, and (**G**) MDA content. Different letters indicate significant differences between groups (*p* < 0.05), while the same letters indicate no significant difference. Error bars were obtained from three biological replicates.

To explore the response of wild jujube to drought stress, one-year-old seedlings from two families with contrasting drought tolerance were exposed to severe drought conditions. Before drought stress treatment, no significant morphological differences were observed between the two families (Figure 1A). Following 15 days of drought stress, the number of leaves showing water loss, yellowing, and abscission increased dramatically in family ‘NO. 5’, with the extent of damage being significantly greater than that in family ‘NO. 1’. In contrast, the ‘NO. 1’ suffered only slight damage and maintained normal growth (Figure 1A).

To investigate the drought tolerance of wild jujube leaves under different drought treatment periods, six physiological indices of the two families were determined. As shown in Figure 1B, compared with the control (0 d), the leaf relative water content (RWC) of both families decreased after drought stress. However, the leaf RWC of ‘NO. 1’ remained higher than that of ‘NO. 5’. After rehydration, the RWC of ‘NO. 1’ could essentially recover to its pre-drought stress level (Figure 1B). Drought stress increased the SOD activity in both families, which peaked after 8 d of treatment. The SOD activity of ‘NO. 1’ was significantly higher than that of ‘NO. 5’ (Figure 1C). Similarly, the contents of SS, SP, and Pro were also higher in ‘NO. 1’ than in ‘NO. 5’ (Figure 1D–F). The difference in its SP content became apparent after 15 d of drought stress, while differences in SS and Pro content were observable as early as 8 d. Furthermore, the MDA content in the leaves of ‘NO. 5’ was significantly higher than that of ‘NO. 1’ after drought stress. Even after 3 days of rehydration, the MDA content in ‘NO. 5’ remained at a relatively high level (Figure 1G).

### 2.2. Changes in Leaf Microstructure and Photosynthetic Parameters of Wild Jujube After Drought Stress

**Figure 2:**
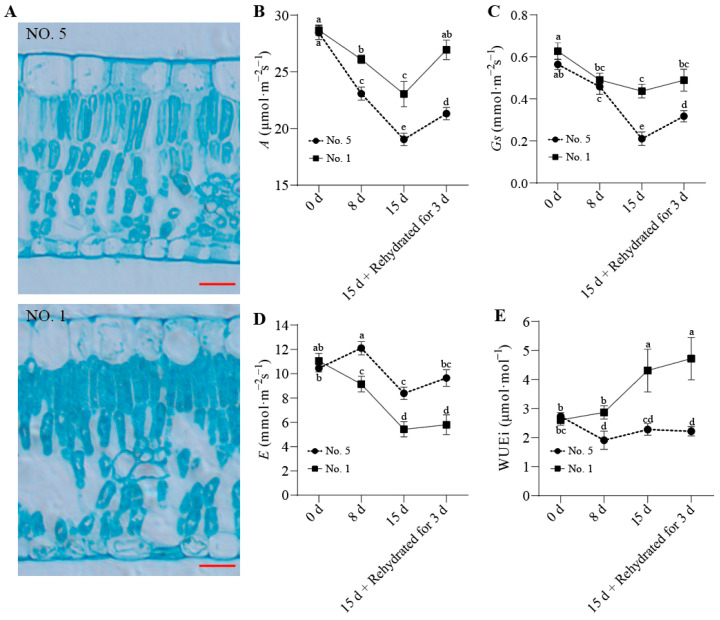
Microscopic structure observation and photosynthetic parameters measurement of *Ziziphus jujuba* var. *spinosa* leaves from two families ‘NO. 1’ and ‘NO. 5’ under drought stress. (**A**) Observation of leaf epidermal cells after 15 d of drought stress. Scale bar: 50 μm, (**B**) photosynthesis (*A*), (**C**) stomatal conductance (*Gs*), (**D**) transpiration (*E*), and (**E**) instantaneous water-use efficiency (WUEi, *A/E*). Different letters indicate significant differences between groups (*p* < 0.05), while the same letters indicate no significant difference. Error bars were obtained from three biological replicates.

To observe the effects of drought stress on the functional properties of wild jujube leaves, epidermal cell microscopic observation and photosynthetic parameter determination were performed. Drought stress caused damage to the leaves of both families, with ‘NO. 5’ exhibiting more severe damage than ‘NO. 1’. The palisade tissue of ‘NO. 5’ appeared loosely arranged, whereas ‘NO. 1’ remained relatively stable and tightly arranged (Figure 2A). As the duration of drought stress increased, the values of photosynthetic rate (*A*) and stomatal conductance (*Gs*) in ‘NO. 5’ declined sharply compared to those of ‘NO. 1’ (Figure 2B,C), while transpiration rate (*E*) values in ‘NO. 5’ remained significantly higher than those of ‘NO. 1’ (Figure 2D). In terms of instantaneous water use efficiency (WUEi), ‘NO. 1’ showed a higher level than ‘NO. 5’ throughout (Figure 2E).

### 2.3. RNA Isolation, Library Construction, and Sequencing

In order to clarify the molecular mechanisms underlying the differences in the drought tolerance of the two analyzed wild jujube families, RNA-seq was performed on the Illumina PE system. The results demonstrated that the extracted total RNA was of sufficient quality and met the experimental conditions. Following the removal of low-quality reads, 41,227,774~46,115,110 clean reads were obtained. The Q30 percentages for the six samples ranged from 93.60% to 95.07%, while the clean read rate was between 96.30% and 99.06% (Table S1). The percentage of the total mapped rate varied from 89.92% to 91.27%, and the unique mapped rate ranged from 78.41% to 79.45% (Table S2). The findings indicated that the data volume satisfied the quality requirements needed for further analysis. Among the three biological replicates of ‘NO. 1’, the correlation coefficients for gene expression levels ranged from a minimum of 0.955 to a maximum of 0.960. Similarly, for the three replicates of ‘NO. 5’, the correlation coefficients ranged from 0.959 to 0.967 (Figure S1). These results demonstrate that the gene expression levels are highly similar and strongly correlated among the three biological replicates within each family.

### 2.4. Analysis of the Differentially Expressed Genes (DEGs)

**Figure 3:**
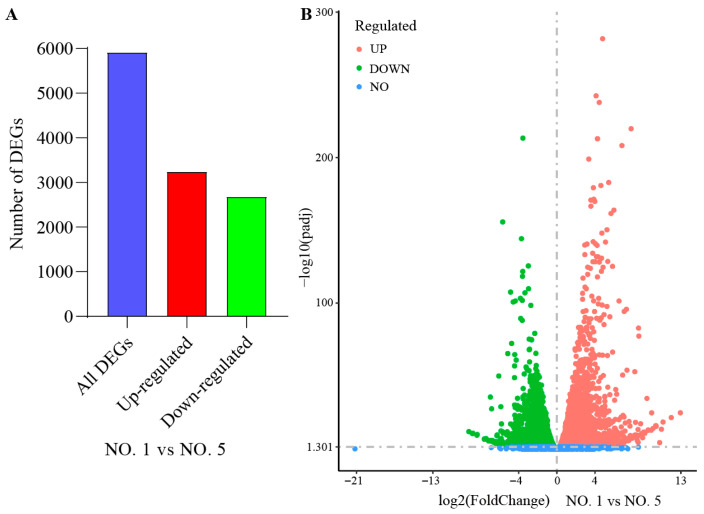
Distribution characterization of DEGs. (**A**) Bar graphs. (**B**) Volcano plots.

DEGs are defined as those that are expressed and have an adjusted FDR < 0.05 and |log2(fold change)| ≥ 1. A total of 5913 genes related to drought stress in leaves were differentially expressed, with 3238 up-regulated genes and 2675 down-regulated genes. Many of these DEGs belong to the AP2/ERF, bZIP, MYB, WRKY, NAC, ZFP, F-box, HSP and bHLH gene families related to drought resistance (Figure 3, Table S3).

**Figure 4:**
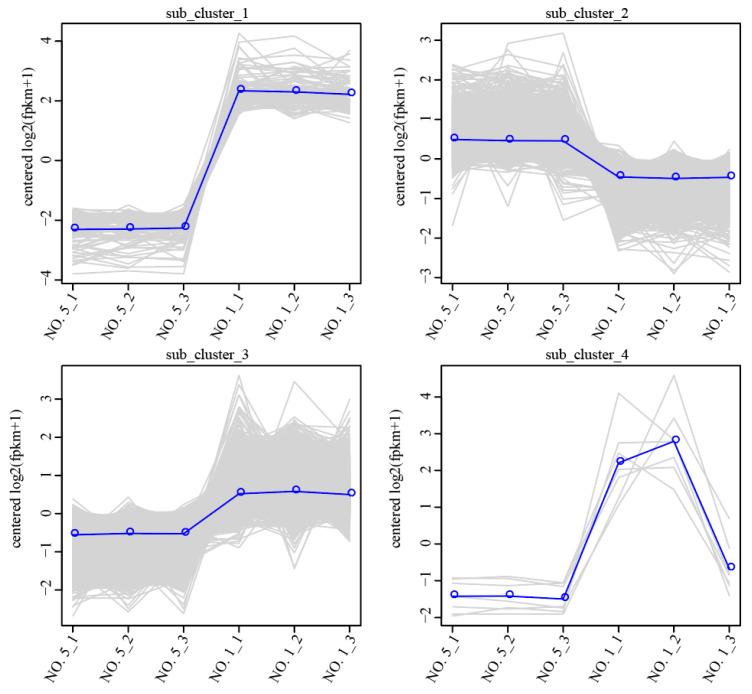
Co-expression trend map of DEGs.

The DEGs were categorized into four clusters, and genes in the same cluster exhibited similar expression level change trends under different treatment conditions (Figure 4, Table S4). The trends of DEGs in different resistant families were classified into four patterns: sharply up-regulated (sub_cluster_1), slowly down-regulated (sub_cluster_2), slowly up-regulated (sub_cluster_3), and first a sharply up-regulated, then down-regulated (sub_cluster_4).

### 2.5. GO Enrichment Analysis of DEGs

**Figure 5:**
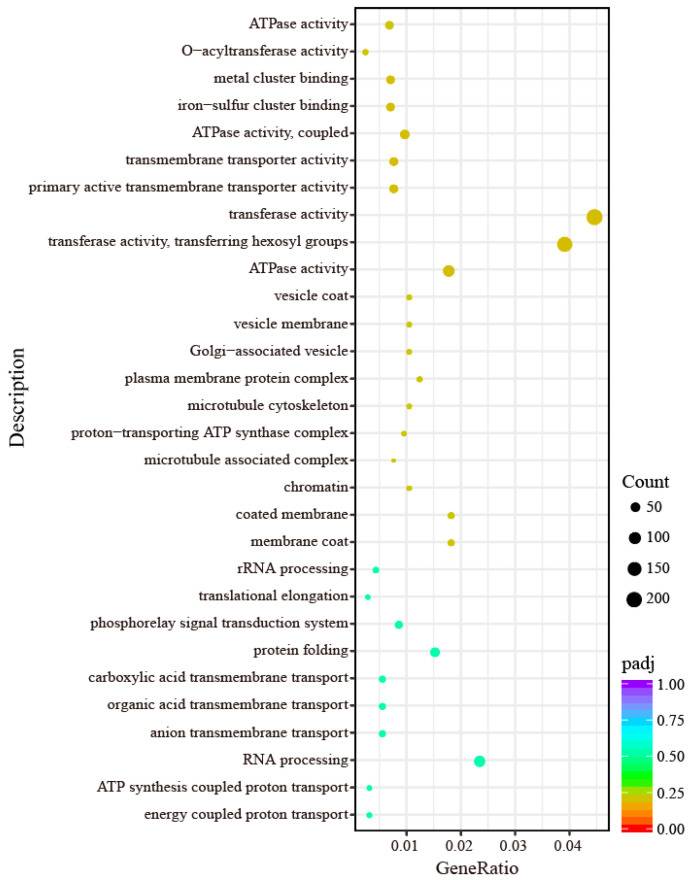
Scatter plot of GO functional annotation for DEGs.

GO functional enrichment and classification analysis was performed on the DEGs between the drought-tolerant family ‘NO. 1’ and the drought-sensitive family ‘NO. 5’. From the GO enrichment analysis results, the top 30 most significant terms were selected and visualized as a scatter plot (Figure 5, Table S5). Within the molecular function (MF) category, the DEGs were mainly enriched in transferase activity, transferring glycosyl groups (221 genes; GO:0016757), transferase activity, transferring hexosyl groups (194 genes; GO:0016758), transmembrane transporter activity (193 genes; GO:0022857), hydrolase activity, acting on acid anhydrides (150 genes; GO:0016817), and hydrolase activity, acting on acid anhydrides, in phosphorus-containing anhydrides (149 genes; GO:0016818). Furthermore, this category includes a large number of functional annotations that are associated with drought stress, e.g., antioxidant activity (35 genes; GO:0016209), protein ubiquitination (21 genes; GO:0016567), protein methylation (9 genes; GO:0006479), protein dephosphorylation (8 genes; GO:0006470), and regulation of cellular protein metabolic process (7 genes; GO:0032268). In the biological process (BP) category, the DEGs were mainly enriched in transmembrane transport (214 genes; GO:0055085), carbohydrate metabolic process (208 genes; GO:0005975), small molecule metabolic process (197 genes; GO:0044281), cell communication (147 genes; GO:0007154), and proteolysis (142 genes; GO:0006508). In addition, there were many classifications related to stimulus and stress response, e.g., response to stress (110 genes; GO:0006950), defense response (33 genes; GO:0006952), response to oxidative stress (28 genes; GO:0006979), cellular response to stress (25 genes; GO:0033554), response to endogenous stimulus (18 genes; GO:0009719), response to hormone (18 genes; GO:0009725), and photosynthesis (18 genes; GO:0015979), and response to external stimulus (7 genes; GO:0009605). In the cellular component (CC) category, the DEGs were mainly enriched in the cytoplasmic part (184 genes; GO:0044444), nucleus (155 genes; GO:0005634), organelle part (109 genes; GO:0044422), intracellular organelle part (109 genes; GO:0044446), and non-membrane-bounded organelle (102 genes; GO:0043228). The differences in DEGs enrichment patterns may reflect the possible biological functional differences in the tolerant and sensitive families in response to drought stress.

**Figure 6:**
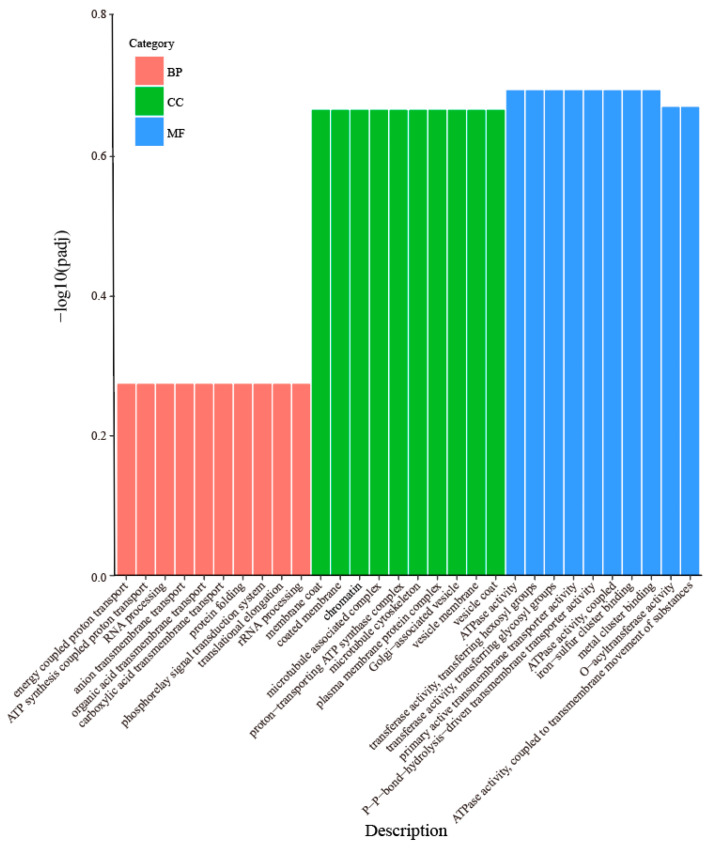
Bar Chart of GO functional annotation for DEGs.

From the GO enrichment analysis results, the top 30 most significant terms were selected and visualized using a bar chart. The results are shown in Figure 6. Within the BP category, DEGs were mainly enriched in energy coupled proton transport (GO:0015985), ATP synthesis coupled proton transport (GO:0015986), RNA processing (GO:0006396), anion transmembrane transport (GO:0098656), and carboxylic acid transmembrane transport (GO:1905039). In the MF category, the DEGs were mainly enriched in ATPase activity (GO:0016887), transferase activity (transferring hexosyl groups) (GO:0016758), transferase activity (transferring glycosyl groups) (GO:0016757), P-P-bond-hydrolysis-driven transmembrane transporter activity (GO:0015405), and primary active transmembrane transporter activity (GO:0015399). In the CC category, the DEGs were mainly enriched in membrane coat (GO:0030117), coated membrane (GO:0048475), chromatin (GO:0000785), microtubule-associated complex (GO:0005875), and proton-transporting ATP synthase complex (GO:0045259) (Table S5). The findings suggested that these functional categories play a core role in the drought stress response in wild jujube.

### 2.6. KEGG Pathway Enrichment Analysis of DEGs

**Figure 7:**
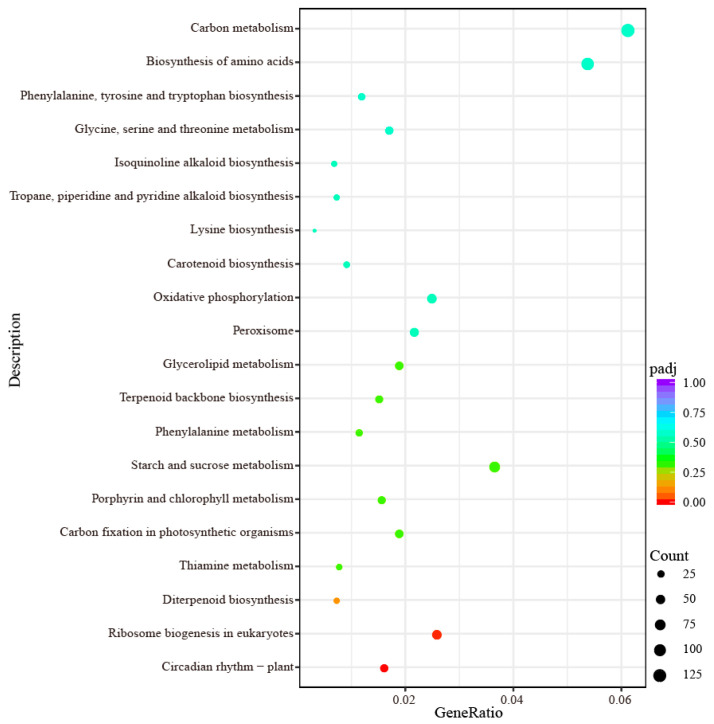
KEGG functional enrichment analysis of DEGs.

The KEGG pathway enrichment analysis was performed on DEGs between the drought-tolerant family ‘NO. 1’ and the drought-sensitive family ‘NO. 5’ of wild jujube to identify the key metabolic pathways in drought stress response. The DEGs were mapped to 121 biological pathways, and 20 of which were significantly enriched (Figure 7). These mainly included carbon metabolism (132 genes; zju01200), biosynthesis of amino acids (116 genes; zju01230), ribosome (108 genes; zju03010), plant-pathogen interaction (94 genes; zju04626), RNA transport (88 genes; zju03013), plant hormone signal transduction (87 genes; zju04075), spliceosome (84 genes; zju03040), protein processing in endoplasmic reticulum (83 genes; zju04141), starch and sucrose metabolism (79 genes; zju00500), glycolysis/gluconeogenesis (62 genes; zju00010), phenylpropanoid biosynthesis (57 genes; zju00940), and ribosome biogenesis in eukaryotes (56 genes; zju03008) (Table S6). Additionally, several metabolic pathways associated with drought stress were identified, including the MAPK signaling pathway (56 genes; zju04016), alkaloid biosynthesis (31 genes; zju00950 and zju00960), and flavonoid biosynthesis (22 genes; zju00941) (Table S6). These pathways are likely to play a crucial role in drought stress of wild jujube.

### 2.7. Validation of DEGs by RT-qPCR

**Figure 8:**
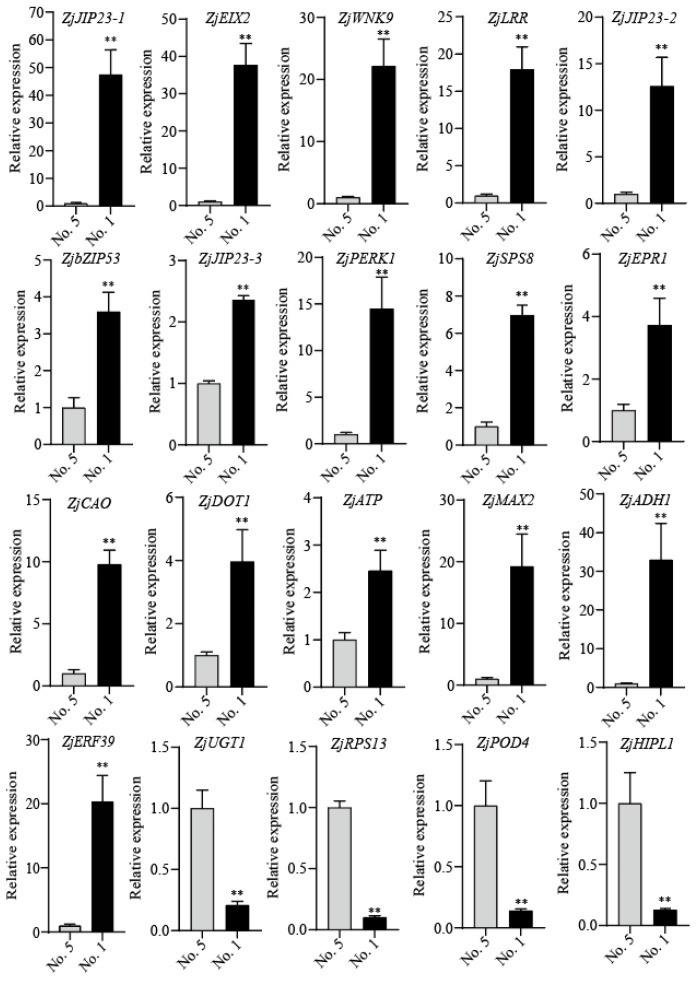
Verification of transcriptome sequencing results and RT-qPCR analysis of DEGs. Error bars were obtained from three biological replicates. The statistical analyses were performed using Student’s *t*-tests (** *p* < 0.01).

To verify the expression profiles of the identified DEGs, 20 genes (16 up-regulated and 4 down-regulated) were selected for an RT-qPCR validation (Figure 8). In the drought-tolerant family ‘NO. 1’ and the drought-sensitive family ‘NO. 5’, *ZjJIP23-1*, *ZjEIX2*, *ZjMAX2*, *ZjWNK9*, *ZjLRR*, *ZjJIP23-2*, *ZjbZIP53*, *ZjJIP23-3*, *ZjPERK1*, *ZjSPS8*, *ZjEPR1*, *ZjCAO*, *ZjDOT1*, *ZjATP*, *ZjADH1* and *ZjERF39* expression levels were significantly up-regulated, whereas *ZjUGT*, *ZjRPS13*, *ZjPOD4* and *ZjHIPL1* expression levels were significantly down-regulated. RT-qPCR results for all 20 genes were consistent with the RNA-seq data, which confirms the reliability and accuracy of the RNA-seq analysis (Table S7).

## 3. Discussion

It is well established that drought stress is a major environmental constraint limiting plant growth, productivity, fruit quality and geographical distribution, with plant drought tolerance governed by a sophisticated regulatory network [17]. Plants have evolved diverse adaptive mechanisms at the physiological, biochemical, cellular and molecular levels to acclimate to water-deficit conditions [27]. However, considerable knowledge gaps still exist regarding the molecular mechanisms of drought tolerance in plants, especially in forest trees [28]. Here, we performed phenotypic observations, physiological index determinations and RNA-seq analyses on the drought-tolerant family ‘NO. 1’ and drought-sensitive family ‘NO. 5’ of wild jujube. By integrating these datasets, we aimed to identify key regulatory genes and unravel the molecular mechanisms underlying drought tolerance in wild jujube. This study lays a critical foundation for the identification of drought-resistant germplasm and the breeding of drought-tolerant wild jujube cultivars.

When soil moisture is insufficient, the main phenotypic symptoms in plants include leaf dehydration, wilting, yellowing and abscission [29]. Following severe drought stress, morphological observations revealed that ‘NO. 1’ exhibited significantly less damage than ‘NO. 5’, maintaining an upright growth habit with greener leaves, which indicated its superior drought tolerance (Figure 1A). RWC is recognized as a key indicator for evaluating drought resistance in plants [30]. Plants with a higher RWC can maintain a relatively stable cellular water status under drought stress, which provides an intact intracellular environment for stress responses and mitigates cellular oxidative damage [31]. Furthermore, the factors determining plant growth and final yield under drought stress include not only the tolerance capacity during stress exposure, but also the ability to recover from drought-induced damage and gradually resume normal growth after the alleviation of water deficit conditions [32]. Under drought stress, ‘NO. 1’ not only had a higher leaf RWC than ‘NO. 5’, but also rapidly restored its leaf water content to the pre-drought level after rehydration, which further confirmed its superior drought resistance (Figure 1B).

In drought-prone environments, plants generate and accumulate large amounts of ROS [33]. Excessive ROS can induce plasma membrane peroxidation, which leads to cell death in severe cases [13]. Meanwhile, this process triggers a lipid peroxidation cascade in the plasma membrane, thereby stimulating the rapid synthesis of SOD in the antioxidant defense system to restore cellular redox homeostasis [34]. Additionally, drought stress causes a decrease in turgor pressure in plant cells due to cellular dehydration. The accumulation of osmotic regulators elevates intracellular solute concentration, reduces osmotic potential, and enhances cellular water retention capacity [17]. SS and SP play pivotal roles in plant osmotic adjustment; their accumulation facilitates cellular water uptake, maintains cell turgor pressure, and stabilizes other biomacromolecules, thus preventing their denaturation or aggregation [35,36]. Pro also acts as a protective osmotic regulator, and its high intracellular concentration can lower cellular water potential while enhancing the ROS-scavenging capacity of antioxidant systems [37]. In contrast, drought stress induces membrane lipid peroxidation, resulting in a significant increase in MDA content that impairs the plant cell membrane system [16]. The results of this experiment were consistent with those of previous studies [38]. Compared with ‘NO. 5’, ‘NO. 1’ exhibited higher SOD activity and higher contents of SS, SP and Pro under drought stress, while its MDA content was relatively lower (Figure 1C–G). This indicates that these substances act synergistically to enhance the drought resistance of wild jujube. Notably, there were differences in the time points at which these osmotic regulators and antioxidant enzymes reached their peak levels. SOD activity, SS content and Pro content increased significantly after 8 d of drought treatment, whereas SP content did not increase significantly until 15 d of treatment. This suggests that the physiological mechanisms underlying the drought resistance of SP possess specific characteristics in wild jujube.

Drought stress impairs plant leaf structure and consequently compromises their photosynthetic performance [39]. Photosynthesis is one of the primary physiological processes involved in plant responses to drought stress [40]. Key parameters, including *A*, *E*, *Gs*, and WUEi, effectively reflect photosynthetic changes in plants under drought stress. *A* is closely correlated with plant growth and development; under drought stress, the decline in soil moisture induces a subsequent reduction in the net photosynthetic rate, making it a theoretically reliable indicator for evaluating plant productivity under water deficit conditions [7]. The results of this study were consistent with those of previous research [29]. Drought stress damaged the leaf structures of both wild jujube families, leading to the reduction in *A*, *Gs* and *E*. However, compared with ‘NO. 5’, ‘NO. 1’ exhibited less leaf structural damage under drought stress, while maintaining relatively higher *A*, *Gs* and *E* levels, along with a significant increase in WUEi (Figure 2). This indicates that ‘NO. 1’ possesses superior self-protective mechanisms that enable it to sustain normal growth under drought conditions.

Transcription factors (TFs) are ubiquitously present in eukaryotes, acting as molecular regulatory switches and playing pivotal roles in the regulatory networks underlying plant drought resistance [41]. They mediate transcriptional regulation by recognizing and binding to specific *cis*-elements in the promoter regions of target genes; additionally, they form protein complexes with other regulatory proteins to activate the expression of downstream target genes and fine-tune gene transcription [42]. To date, the TF families identified to be involved in plant drought resistance responses include AP2/ERF, MYB, bZIP, HSF, bHLH, NAC and WRKY [19]. In this study, members of these TF families were also found to be significantly differentially expressed in the drought-tolerant family ‘NO. 1’ (Figure 3, Table S3). RT-qPCR validation further confirmed that *ZjbZIP53* and *ZjERF39* were drought-induced and up-regulated in ‘NO. 1’ (Figure 8). Beyond TFs, several functional proteins also play crucial roles in plant drought stress responses. In Arabidopsis, Alcohol dehydrogenase 1 (*ATADH1*) and the ATP synthase small subunit gene (*AtMtATP6*) improve seedling survival rates and enhance plant drought tolerance [43,44]. In maize (*Zea mays*), members of the Serine Peptidase S8 family (ZmSPS8s) are significantly induced in drought-tolerant inbred lines [45]. In the screening of drought-resistant genes in *Arachis duranensis*, the glycine-rich protein DOT1-like gene is highly expressed under drought stress [46]. Integrating physiological indices and transcriptome data of *Jatropha curcas* under drought stress revealed that chlorophyll a oxygenase (CAO) is significantly up-regulated in plant leaves under severe drought, and its expression level recovers to the control level after rewatering [47]. In the present study, the homologous genes *ZjADH1*, *ZjATP*, *ZjSPS8*, *ZjDOT1* and *ZjCAO* in wild jujube were also significantly drought-induced (Figure 8). The identification of these candidate genes lays a solid research foundation for subsequent gene functional validation and the elucidation of the molecular mechanisms underlying wild jujube drought tolerance.

Highly conserved mitogen-activated protein kinases (MAPKs) are key mediators of intracellular and extracellular signal transduction in eukaryotes [48]. When plants are exposed to drought stress, MAPKs act as osmotic signal transducers to mediate the activation of downstream TFs, thereby driving plant adaptive responses to water-deficit conditions [49]. In this study, numerous DEGs were significantly enriched in the MAPK signaling pathway and transmembrane transport pathways (Figure 7, Table S6), suggesting that these genes may be transcriptionally induced during the early water loss signal response in wild jujube. Phytohormones also play a pivotal role in orchestrating plant drought stress responses [50]. Previous studies have demonstrated that abscisic acid (ABA), jasmonic acid (JA), indole-3-acetic acid (IAA), gibberellin (GA), cytokinin (CTK) and salicylic acid (SA) crosstalk with each other to synergistically regulate multiple signaling cascades, which constitutes a core regulatory mechanism underlying the development of plant drought tolerance [51]. Notably, exogenous JA application has been shown to significantly enhance the activity of various antioxidant enzymes in plants under drought stress [52]. In the present study, a large number of DEGs were annotated to the plant hormone signal transduction pathway (Figure 7, Table S6). RT-qPCR validation further revealed that three genes encoding jasmonate-induced protein-like proteins in wild jujube, *ZjJIP23-1*, *ZjJIP23-2* and *ZjJIP23-3*, were all significantly up-regulated in the drought-tolerant family ‘NO. 1’ (Figure 8). This implies that JA may play a critical regulatory role in the drought resistance of wild jujube, while whether JA crosstalks with other phytohormones or signaling molecules to modulate drought tolerance requires further experimental validation.

Carbohydrate synthesis and metabolism represent a pivotal metabolic process that supplies the sugars and energy essential for sustaining cellular physiological activities [53]. Drought stress can induce altered expression patterns of genes involved in this metabolic pathway, which in turn contributes to plant adaptation to water-deficit conditions [54]. Furthermore, the enrichment of carbohydrate synthesis and metabolism pathways has been reported in *Arabidopsis*, maize and onion under drought stress, indicating that this may represent a conserved regulatory mechanism for enhancing plant drought resistance [35,55]. Amino acid biosynthesis and metabolism not only generate SP that play critical roles in mediating plant drought stress responses but also provide fundamental components for structural and functional proteins encoded by genes [36]. In the present study, a large number of DEGs were significantly enriched in the carbohydrate biosynthesis and amino acid biosynthesis pathways; additionally, several of these DEGs were associated with antioxidant activity and photosynthesis-related signaling pathways (Tables S5 and S6). These findings from DEG analysis are consistent with the results obtained from physiological indicator measurements.

In response to drought stress, the genetic regulatory mechanisms in plants also involve the orchestration of complex metabolic processes, including the de novo synthesis of beneficial secondary metabolites in cells [55]. These secondary metabolites enhance plant defense systems by conferring diverse biological activities, such as protecting plants against oxidative stress, and are thus defined as stress-resistant metabolites [56]. Many secondary metabolites, including flavonoids, alkaloids and betaines, serve as valuable dietary components and medicinal agents; notably, these compounds are synthesized and exert potent antioxidant effects in plants under drought stress, which also reflects the important medicinal value of *Z. jujuba* var. *spinosa* [3,7]. Flavonoids play a pivotal role in mediating plant responses to drought stress [57]. Previous studies have reported that flavonoids exert a protective effect against drought stress through ROS detoxification [58]. Numerous studies have also demonstrated that genes involved in flavonoid biosynthesis are independently regulated by drought stress signals [59,60]. Alkaloids are nitrogen-containing secondary metabolites with important ecological functions and high medicinal value [61]. Drought stress can induce comprehensive changes in various aspects of the alkaloid profile, and these effects are mediated via a sophisticated regulatory network involving physiological modulation, enzyme activity regulation, transcriptional factor-mediated control and epigenetic modification [62]. In the present study, a large number of DEGs were significantly enriched in the flavonoid and alkaloid biosynthesis pathways (Figure 7, Table S6), which will be the focus of our follow-up research (for example, trans-cinnamate 4-monooxygenase, chalcone synthase and dihydroflavonol 4-reductase). In summary, an integrated analysis of physiological and transcriptomic data revealed that these key candidate DEGs coordinately regulate the drought stress response in the leaves of *Z. jujuba* var. *spinosa* through the crosstalk of osmotic adjustment, antioxidant systems, photosynthetic regulation, phytohormone signaling and other metabolic pathways, in conjunction with the transcriptional regulatory network.

## 4. Materials and Methods

### 4.1. Plant Materials and Treatment

The experimental materials consisted of one-year-old seedlings from two *Z. jujuba* var. *spinosa* families, obtained from the National Forest Germplasm Resource Repository for Wild Jujube at Shenyang Agricultural University (located in Kazuo County, Chaoyang, Liaoning, China; longitude 119°49′48″ E, latitude 41°33′53″ N). Both families are indigenous to Kazuo County. Healthy seedlings were individually potted in plastic pots (top diameter 17.0 cm, bottom diameter 12.0 cm, height 17.0 cm). Drought treatment was implemented by withholding water for 8 and 15 d, with 0 d set as the control (soil moisture content: 40~45%) [63]. Rehydration was conducted on the 16th d, and phenotypic changes at each time point were observed and recorded on the 3rd d post-rehydration. Mature leaves were sampled for the determination of physiological and photosynthetic indicators, while other leaves were reserved for transcriptome sequencing. Three biological replicates were prepared for each family to support subsequent analyses. Upon being transported back to the laboratory, all samples were stored in an ultra-low temperature freezer (−80 °C) for subsequent RNA extraction. The experimental group (drought-tolerant family) included NO. 1_1 (GSM5462957), NO. 1_2 (GSM5462958), and NO. 1_3 (GSM5462959), whereas the control group (drought-sensitive family) comprised NO. 5_1 (GSM5462960), NO. 5_2 (GSM5462961), and NO. 5_3 (GSM5462962).

### 4.2. Physiological Analysis

RWC determination was conducted following the method described by Liu et al., with three biological replicates for each sample [29]. Mature leaves were detached from each twig of soil-grown seedlings, weighed immediately to record the fresh weight (FW). Subsequently, the leaves were floated in distilled water for 24 h, and their turgid weight (TW) was measured. They were then oven-dried at 105 °C for 8 h until a constant weight was achieved, and the dry weight (DW) was recorded. Leaf RWC was calculated using the formula: (FW − DW)/(TW − DW) × 100%.

SOD activity was assayed via the nitroblue tetrazolium (NBT) method, referring to the protocol by Liu et al. with three replicates per measurement [38]. SS concentration was determined using the anthrone colorimetric method [45], while SP concentration was quantified by the Coomassie Brilliant Blue G-250 staining method. Pro content was measured following the method described by Liu et al., with all determinations performed in triplicate [64]. MDA content was estimated based on the thiobarbituric acid (TBA) reactive substances assay [65].

### 4.3. Microscopic Structure Observation

For anatomical observation, leaves were cut into small square segments (approximately 0.5 cm × 0.5 cm). These segments were immersed in FAA fixative for 24 h, decolorized with alcohol, embedded in paraffin, and stained with safranin and malachite green. The structural characteristics of the leaves were then observed using a fluorescence inverted microscope.

### 4.4. Photosynthetic Parameter Measurement

A LI-COR 6400 portable photosynthesis system was employed to determine net photosynthetic rate (*A*), stomatal conductance (*Gs*), and transpiration rate (*E*) of soil-grown seedlings. Mature leaves were selected for testing, as they exhibit fully developed stomata and relatively stable photosynthetic parameters [29]. Instantaneous water use efficiency (WUEi) was calculated as the ratio of *A*/*E*. All determinations were carried out with three independent biological replicates.

### 4.5. RNA Extraction and Transcriptome Data Analysis

Transcriptome sequencing was conducted on leaf samples (withholding water for 15 d) from the two *Z. jujuba* var. *spinosa* families, with three biological replicates. Approximately 1.0 g of leaves was fully ground in liquid nitrogen, followed by the addition of Trizol reagent; total plant RNA was then extracted via the CTAB method [66]. RNA integrity was evaluated using the RNA Nano 6000 Assay Kit on the Bioanalyzer 2100 System (Agilent Technologies, Santa Clara, CA, USA). Each sample had total RNA concentrations ranging from 183 ng/μL to 291 ng/μL; total RNA yield ranged from 6.41 ng to 10.19, the RIN value ranged from 5.3 to 7.5, and all test results achieved grade A (Table S8). cDNA libraries were sequenced with the TruSeq PE Cluster Kit v3-cBot-HS (Illumina Inc., San Diego, CA, USA), generating 150 bp paired-end reads through sequencing-by-synthesis technology. Using the *Z. jujuba* genome as the reference [67], sequence alignment and subsequent analyses were performed. Hisat2 v2.0.5 software was utilized to map RNA-Seq reads to the reference genome. Raw image data generated from high-throughput sequencing were converted into sequence reads via Illumina CASAVA 1.8, while the R programming language (version 4.0.2) was adopted for raw data filtration. Following the removal of adapter sequences, reads containing over 10% ambiguous bases, reads with unknown nucleotides and low-quality reads (quality score < 30), high-quality clean reads were obtained. Additionally, the gene read counts were normalized using the fragments per kilobase of exon per million mapped fragments (FPKM) method. The FPKM method was employed to simultaneously eliminate the confounding effects of gene length and sequencing depth on gene expression levels, thereby allowing accurate comparison and standardized quantification across different genes and samples. The RNA-seq and assembly work was undertaken by Novogene Technology Co., Ltd. (Sacramento, CA, USA).

### 4.6. Identification of Differentially Expressed Genes (DEGs)

DEGs were identified via the DESeq program (version 1.20.0), which was utilized to construct a DataSet object for the normalization of input matrix data [68]. The resulting *p*-values were adjusted by the Benjamini and Hochberg approach to control the false discovery rate (FDR). Genes with an FDR < 0.05 and a |log2(fold change)| ≥ 1 were defined as significantly DEGs.

### 4.7. GO and KEGG Enrichment Analysis

Gene Ontology (GO) enrichment analysis was implemented with the clusterProfiler R package (version 3.4.4), and a corrected *p*-value < 0.05 was set as the threshold for defining DEGs with significantly enriched GO terms. The Kyoto Encyclopedia of Genes and Genomes (KEGG) is a database resource designed to elucidate the high-level functions and characteristics of biological systems (http://www.genome.jp/kegg/, accessed on 13 March 2026) [65]. Additionally, the clusterProfiler R package was employed to conduct enrichment analysis of DEGs in KEGG pathways.

### 4.8. Reverse Transcriptase–Quantitative PCR (RT-qPCR)

Twenty genes were selected for RT-qPCR analysis to verify the reliability of transcriptome sequencing results. *ZjActin* was used as the internal reference gene, which guarantees the accuracy and dependability of quantifying the expression level of the target gene [69]. Gene-specific primers were designed via Primer Premier 5.0, and their sequences are provided in Table 1. NO. 5 under 15 d of drought stress was selected as the control group sample, and NO. 1 as the experimental group sample. Approximately 1.0 μg of total RNA was reverse-transcribed to synthesize first-strand cDNA using the EasyScript One-Step gDNA Removal and cDNA Synthesis SuperMix (TransGen, China). RT-qPCR assays were conducted on the StepOne Real-Time PCR System with SuperReal PreMix Plus SYBR Green (TransGen, Beijing, China). The PCR reaction protocol was composed of the following procedures: an initial denaturation at 95 °C for 3 min, followed by 40 cycles of 95 °C for 10 s, 60 °C for 15 s, and 60 °C for 1 min, and concluding with 95 °C for 15 s. Subsequent to the amplification protocol, a melting curve was established to verify the specificity of each primer pair. The 2^−ΔΔCT^ method was adopted to calculate the relative gene expression levels [70]. This analysis included three independent biological replicates.

### 4.9. Statistical Analysis

Data processing and statistical analysis were performed using Microsoft Excel and SPSS v26.0 software, respectively. Bar graphs were generated with GraphPad Prism v9.5. The experimental data were presented as mean ± standard deviation (SD). The significance of differences between treatments was assessed by employing Student’s *t*-tests and one/two-way ANOVA.

## 5. Conclusions

This study comprehensively explores the drought tolerance mechanisms of wild jujube through an integrated analysis of phenotypic, physiological, and transcriptomic data, using two distinct wild jujube families. The results collectively confirm that significant differences in drought tolerance exist between the two families, with the ‘NO. 1’ exhibiting superior drought resistance compared to the ‘NO. 5’. Therefore, ‘NO. 1’ is recommended as a priority material for future studies on drought-resistant molecular mechanisms, variety breeding, and extension. RNA-seq revealed that a large number of DEGs were significantly up-regulated and involved in drought stress signaling pathways, plant hormone signal transduction, and secondary metabolite biosynthesis. These key candidate genes can be further subjected to genetic transformation to verify their functional roles and assess their adaptability to environmental fluctuations. Alternatively, they may serve as molecular markers for introgression breeding or early screening and characterization of drought-resistant germplasm resources. Collectively, these findings offer a theoretical basis for in-depth investigations into the complex regulatory mechanisms underlying drought response in wild jujube and other economic forest species.

## Figures and Tables

**Table 1:** Sequence information of the primers.

Genes	Forward Primer	Reverse Primer
*ZjJIP23-1* (107421448)	ATGACATTATGCTGGCTTGG	CTTCTGGCTTATTAAAGTAGTC
*ZjEIX2* (107414618)	TTACAGGACTCATCGGTTTA	TTGGGACAGGCTAGAAGG
*ZjMAX2* (107404539)	TATGCTCTTACGGCTCCCTCTG	TTCAAGCTGCCAATTCCA
*ZjWNK9* (107423206)	CCCTCACCTAAGACAACC	AACCGAGATAATTGGAGTAA
*ZjLRR* (107426956)	TGGTCGTGTTTACCCATTA	ATCCACCGTCATCAGCAA
*ZjJIP23-2* (107416210)	TATGGCAATGGAATCTCG	ATCTTGGCAGGGTATGGA
*ZjbZIP53* (107418952)	TAGCGAAACGACTTGTAG	CTGGACCATTCTCATCTT
*ZjJIP23-3* (107433945)	ACCCTTCTGATTCTTCCC	AACCCATTCCATTGACTTTC
*ZjPERK1* (107427329)	GATGGCAAACCAGTAGCA	CAACGCAGACACCGAGTA
*ZjSPS8* (107422854)	CCAGTTCTGCCTAAACCAAAG	GGCTTGTAGTAAGGTGGTGGC
*ZjEPR1* (107422855)	ATGTCTTCCTCGTATTTGC	GGTTGTGATATGGGTTTGG
*ZjCAO* (107421073)	CAACAGATGGTAAATGCGAGAA	AGGAAGGGTGGCAGATGG
*ZjDOT1* (112493315)	AGAAAGAACTACATGCAATAC	GAGACCATACAGCGAGCA
*ZjATP* (107429402)	TTGCCGACTACGCTACCT	AGTGATCTTCCCACCCTTT
*ZjADH1* (107435988)	CACCACCACAGCATAACG	CAATACCTCCTGCCTCAT
*ZjERF39* (107409581)	GTTTGGCTCGGCTCATTC	GGCGGAGTTTCCTTTCAG
*ZjUGT* (107406327)	CAACTCCCTTCGGCTCTT	CAGTGGCAGTCCTGGTAA
*ZjRPS13* (112489358)	TCGTATTGGAGGAGTGGA	AGAGTATTTGCCGAGACC
*ZjPOD4* (107411607)	ACAATGGGATGTGAAACT	AAGTAGGACCTGGGAGTT
*ZjHIPL1* (107418502)	TTCAAGGAGCATTACCAG	TTCACCAGCGAAACATAC
*ZjActin*	AGCCTTCCTGCCAACGAGT	TTGCTTCTCACCCTTGATGC

## Data Availability

The data that support the findings of this study, including the raw RNA-seq, is openly available at NCBI (https://www.ncbi.nlm.nih.gov/geo/query/acc.cgi?acc=GSE180449, accessed on 13 March 2026), Gene Expression Omnibus (GEO) number: GSE180449.

## Supplementary Materials

The following supporting information can be downloaded at: https://www.mdpi.com/article/10.3390/ijms27062669/s1.

## References

[1] Ruan W., Liu J., Zhang S., Huang Y., Zhang Y., Wang Z. (2024). Sour Jujube (*Ziziphus jujuba* var. *Spinosa*): A Bibliometric Review of Its Bioactive Profile, Health Benefits and Trends in Food and Medicine Applications. Foods.

[2] Xue T.-T., Ruan K.-H., Xu H.-B., Liu H.-B., Tang Z.-S., Yang Y.-G., Duan J.-A., Sun X.-X., Wang M., Song Z.-X. (2024). Effect of Different Drying Methods on the Drying Characteristics, Chemical Properties and Antioxidant Capacity of *Ziziphus jujuba* var. *Spinosa* Fruit. LWT.

[3] Hua Y., Xu X., Guo S., Xie H., Yan H., Ma X., Niu Y., Duan J.-A. (2022). Wild Jujube (*Ziziphus jujuba* var. *Spinosa*): A Review of Its Phytonutrients, Health Benefits, Metabolism, and Applications. J. Agric. Food Chem..

[4] Wang D., Ho C.-T., Bai N. (2022). *Ziziphi Spinosae Semen*: An Updated Review on Pharmacological Activity, Quality Control, and Application. J. Food Biochem..

[5] Li B., Zhang Y., Kang Y., Wang Y., Liu R., Liu Q., Dong S. (2024). Physiological Response to Low-Temperature Stress and Cold Resistance Evaluation of *Ziziphus jujuba* var. *Spinosa* Clones from Different Provenances. Forests.

[6] Li M., Zhang C., Hou L., Yang W., Liu S., Pang X., Li Y. (2021). Multiple Responses Contribute to the Enhanced Drought Tolerance of the Autotetraploid *Ziziphus jujuba* Mill. var. *Spinosa*. Cell Biosci..

[7] Song F., Yang Q., Huang J., Guo Z., Li Y., Deng W. (2026). Plant Drought Stress: Physiological, Biochemical and Molecular Mechanisms. Plant Stress.

[8] Jarin A.S., Islam M.M., Rahat A., Ahmed S., Ghosh P., Murata Y. (2024). Drought Stress Tolerance in Rice: Physiological and Biochemical Insights. Int. J. Plant Biol..

[9] Bhusal N., Lee M., Lee H., Adhikari A., Han A., Han A., Kim H.S. (2021). Evaluation of Morphological, Physiological, and Biochemical Traits for Assessing Drought Resistance in Eleven Tree Species. Sci. Total Environ..

[10] Mohammadkhani N., Heidari R. (2008). Drought-Induced Accumulation of Soluble Sugars and Proline in Two Maize Varieties. World Appl. Sci. J.

[11] Szabados L., Savouré A. (2010). Proline: A Multifunctional Amino Acid. Trends Plant Sci..

[12] Wang M., Han T., Hua T., Liu R., Feng Y., Sun Z., Zhang Y., Sun X. (2026). MdCoL Regulating Columnar Growth in Apple Trees Positively Mediates Drought Stress Responses. Plant Physiol. Biochem..

[13] Qi J., Song C.-P., Wang B., Zhou J., Kangasjärvi J., Zhu J.-K., Gong Z. (2018). Reactive Oxygen Species Signaling and Stomatal Movement in Plant Responses to Drought Stress and Pathogen Attack. J. Integr. Plant Biol..

[14] Chen D., Wang S., Cao B., Cao D., Leng G., Li H., Yin L., Shan L., Deng X. (2016). Genotypic Variation in Growth and Physiological Response to Drought Stress and Re-Watering Reveals the Critical Role of Recovery in Drought Adaptation in Maize Seedlings. Front. Plant Sci..

[15] Paes De Melo B., Carpinetti P.D.A., Fraga O.T., Rodrigues-Silva P.L., Fioresi V.S., De Camargos L.F., Ferreira M.F.D.S. (2022). Abiotic Stresses in Plants and Their Markers: A Practice View of Plant Stress Responses and Programmed Cell Death Mechanisms. Plants.

[16] Fei X., Li J., Kong L., Hu H., Tian J., Liu Y., Wei A. (2020). miRNAs and Their Target Genes Regulate the Antioxidant System of Zanthoxylum Bungeanum under Drought Stress. Plant Physiol. Biochem..

[17] Mansoor S., Chung Y.S. (2024). Functional Phenotyping: Understanding the Dynamic Response of Plants to Drought Stress. Curr. Plant Biol..

[18] Li L.J., Li J.Y., Hao L.H., Yi H.L. (2026). VvWRKY70, a newly identified grape WRKY transcription factor, confers drought tolerance via coordinated physiological and molecular responses. Plant Sci..

[19] Liu F., Xi M., Liu T., Wu X., Ju L., Wang D. (2024). The Central Role of Transcription Factors in Bridging Biotic and Abiotic Stress Responses for Plants’ Resilience. New Crops.

[20] Cao L., Lu X., Wang G., Zhang P., Fu J., Wang Z., Wei L., Wang T. (2021). Transcriptional Regulatory Networks in Response to Drought Stress and Rewatering in Maize (*Zea mays* L.). Mol. Genet. Genom..

[21] Nakashima K., Yamaguchi-Shinozaki K., Shinozaki K. (2014). The Transcriptional Regulatory Network in the Drought Response and Its Crosstalk in Abiotic Stress Responses Including Drought, Cold, and Heat. Front. Plant Sci..

[22] Tian J.W., Yuan P.H., Gao X., Wang H.T., Wang M.M., Jiao J., Zhang K.X., Hao P.B., Song C.H., Zheng X.B. (2025). The AP2/ERF transcription factor MhERF113-like positively regulates drought tolerance in transgenic tomato and apple. Plant Physiol. Biochem..

[23] Wei H., Wang X., Wang K.T., Tang X., Zhang N., Si H.J. (2024). Transcription factors as molecular switches regulating plant responses to drought stress. Physiol. Plant..

[24] Choi J., Lim C.W., Lee S.C. (2025). Role of pepper bZIP transcription factor CaADBZ1 in abscisic acid signalling and drought stress response. Physiol. Plant..

[25] de la Fuente G.C.M., Bullones A., Llano Y.P., González D.M., Batista-Garcia R.A., Claros M.G., Fernandez-Pozo N., Fernández-Ocaña A.M. (2026). Transcriptome Profiling Reveals Divergent Response Strategies in Two Olive Cultivars with Contrasting Drought Tolerance. Plant Physiol..

[26] Wei X., Cao K., Lu X., Lu R., Li L., Huang R., He Y., Chen J., Xiao J. (2026). Integrative Physiological and Transcriptomic Analysis Reveals Drought Response Mechanisms in *Abrus mollis* Hance. BMC Plant Biol..

[27] Rasheed A., Li H., Tahir M.M., Mahmood A., Nawaz M., Shah A.N., Aslam M.T., Negm S., Moustafa M., Hassan M.U. (2022). The Role of Nanoparticles in Plant Biochemical, Physiological, and Molecular Responses under Drought Stress: A Review. Front. Plant Sci..

[28] Carraro E., Di Iorio A. (2022). Eligible Strategies of Drought Response to Improve Drought Resistance in Woody Crops: A Mini-Review. Plant Biotechnol. Rep..

[29] Liu Q., Wang Z., Yu S., Li W., Zhang M., Yang J., Li D., Yang J., Li C. (2021). Pu-miR172d Regulates Stomatal Density and Water-Use Efficiency via Targeting *PuGTL1* in Poplar. J. Exp. Bot..

[30] Zhang Q., Liu H., Wu X., Wang W. (2020). Identification of Drought Tolerant Mechanisms in a Drought-Tolerant Maize Mutant Based on Physiological, Biochemical and Transcriptomic Analyses. BMC Plant Biol..

[31] Jiang Y., Su S., Chen H., Li S., Shan X., Li H., Liu H., Dong H., Yuan Y. (2023). Transcriptome Analysis of Drought-Responsive and Drought-Tolerant Mechanisms in Maize Leaves under Drought Stress. Physiol. Plant..

[32] Li S., Lin Y.-C.J., Wang P., Zhang B., Li M., Chen S., Shi R., Tunlaya-Anukit S., Liu X., Wang Z. (2019). The AREB1 Transcription Factor Influences Histone Acetylation to Regulate Drought Responses and Tolerance in *Populus trichocarpa*. Plant Cell.

[33] Avramova V., AbdElgawad H., Vasileva I., Petrova A.S., Holek A., Mariën J., Asard H., Beemster G.T.S. (2017). High Antioxidant Activity Facilitates Maintenance of Cell Division in Leaves of Drought Tolerant Maize Hybrids. Front. Plant Sci..

[34] Mansoor S., Ali Wani O., Lone J.K., Manhas S., Kour N., Alam P., Ahmad A., Ahmad P. (2022). Reactive Oxygen Species in Plants: From Source to Sink. Antioxidants.

[35] Jiang C., Li X., Zou J., Ren J., Jin C., Zhang H., Yu H., Jin H. (2021). Comparative Transcriptome Analysis of Genes Involved in the Drought Stress Response of Two Peanut (*Arachis hypogaea* L.) Varieties. BMC Plant Biol..

[36] Zhang J., Huang D., Zhao X., Zhang M. (2021). Evaluation of Drought Resistance and Transcriptome Analysis for the Identification of Drought-Responsive Genes in *Iris germanica*. Sci. Rep..

[37] Ghosh U.K., Islam M.N., Siddiqui M.N., Khan M.A.R. (2021). Understanding the Roles of Osmolytes for Acclimatizing Plants to Changing Environment: A Review of Potential Mechanism. Plant Signal. Behav..

[38] Chen L., Li C., Zhang J., Li Z., Zeng Q., Sun Q., Wang X., Zhao L., Zhang L., Li B. (2024). Physiological and Transcriptome Analyses of Chinese Cabbage in Response to Drought Stress. J. Integr. Agric..

[39] Zahra N., Hafeez M.B., Kausar A., Al Zeidi M., Asekova S., Siddique K.H.M., Farooq M. (2023). Plant Photosynthetic Responses under Drought Stress: Effects and Management. J. Agron. Crop Sci..

[40] Huang Q., Zhang M., Li C., Li B., Zhuo S., Yang Y., Chen Y., Zhong A., Liu H., Lai W. (2025). Response Mechanism of Water Status and Photosynthetic Characteristics of Cotoneaster Multiflorus under Drought Stress and Rehydrated Conditions. Front. Plant Sci..

[41] Hu Y., Chen X., Shen X. (2022). Regulatory Network Established by Transcription Factors Transmits Drought Stress Signals in Plant. Stress Biol..

[42] Wei M., Liu Q., Wang Z., Yang J., Li W., Chen Y., Lu H., Nie J., Liu B., Lv K. (2020). PuHox52-mediated Hierarchical Multilayered Gene Regulatory Network Promotes Adventitious Root Formation in *Populus ussuriensis*. New Phytol..

[43] Shi H., Liu W., Yao Y., Wei Y., Chan Z. (2017). Alcohol Dehydrogenase 1 (ADH1) Confers Both Abiotic and Biotic Stress Resistance in *Arabidopsis*. Plant Sci..

[44] Zhang X., Liu S., Takano T. (2008). Overexpression of a Mitochondrial ATP Synthase Small Subunit Gene (AtMtATP6) Confers Tolerance to Several Abiotic Stresses in *Saccharomyces cerevisiae* and *Arabidopsis thaliana*. Biotechnol. Lett..

[45] Cui H., Zhou G., Ruan H., Zhao J., Hasi A., Zong N. (2023). Genome-Wide Identification and Analysis of the Maize Serine Peptidase S8 Family Genes in Response to Drought at Seedling Stage. Plants.

[46] Carmo L.S.T., Martins A.C.Q., Martins C.C.C., Passos M.A.S., Silva L.P., Araujo A.C.G., Brasileiro A.C.M., Miller R.N.G., Guimarães P.M., Mehta A. (2019). Comparative Proteomics and Gene Expression Analysis in *Arachis duranensis* Reveal Stress Response Proteins Associated to Drought Tolerance. J. Proteom..

[47] Sapeta H., Lourenço T., Lorenz S., Grumaz C., Kirstahler P., Barros P.M., Costa J.M., Sohn K., Oliveira M.M. (2016). Transcriptomics and Physiological Analyses Reveal Co-Ordinated Alteration of Metabolic Pathways in *Jatropha curcas* Drought Tolerance. J. Exp. Bot..

[48] Couto D., Zipfel C. (2016). Regulation of Pattern Recognition Receptor Signalling in Plants. Nat. Rev. Immunol..

[49] Manna M., Rengasamy B., Sinha A.K. (2023). Revisiting the Role of MAPK Signalling Pathway in Plants and Its Manipulation for Crop Improvement. Plant Cell Environ..

[50] Huang S., Jin S. (2025). Enhancing Drought Tolerance in Horticultural Plants through Plant Hormones: A Strategic Coping Mechanism. Front. Plant Sci..

[51] Liao Z., Chen B., Boubakri H., Farooq M., Mur L.A.J., Urano D., Teo C.H., Tan B.C., Hasan M.M., Aslam M.M. (2025). The Regulatory Role of Phytohormones in Plant Drought Tolerance. Planta.

[52] Alam M.M., Nahar K., Hasanuzzaman M., Fujita M. (2014). Exogenous Jasmonic Acid Modulates the Physiology, Antioxidant Defense and Glyoxalase Systems in Imparting Drought Stress Tolerance in Different Brassica Species. Plant Biotechnol. Rep..

[53] Huang X., Guo W., Yang L., Zou Z., Zhang X., Addo-Danso S.D., Zhou L., Li S. (2023). Effects of Drought Stress on Non-Structural Carbohydrates in Different Organs of *Cunninghamia lanceolata*. Plants.

[54] Wang J., Gao X., Wang X., Song W., Wang Q., Wang X., Li S., Fu B. (2022). Exogenous Melatonin Ameliorates Drought Stress in Agropyron Mongolicum by Regulating Flavonoid Biosynthesis and Carbohydrate Metabolism. Front. Plant Sci..

[55] Ahmad S., Belwal V., Punia S.S., Ram M., Dalip, Rajput S.S., Kunwar R., Meena M.K., Gupta D., Kumawat G.L. (2023). Role of Plant Secondary Metabolites and Phytohormones in Drought Tolerance: A Review. Gesunde Pflanz..

[56] Ghasemi S., Kumleh H.H., Kordrostami M., Rezadoost M.H. (2023). Drought Stress-Mediated Alterations in Secondary Metabolites and Biosynthetic Gene Expression in Cumin Plants: Insights from Gene-Specific and Metabolite-Level Analyses. Plant Stress.

[57] Rao M.J., Duan M., Eman M., Yuan H., Sharma A., Zheng B. (2024). Comparative Analysis of Citrus Species’ Flavonoid Metabolism, Gene Expression Profiling, and Their Antioxidant Capacity under Drought Stress. Antioxidants.

[58] Alizadeh Yeloojeh K., Saeidi G., Sabzalian M.R. (2020). Drought Stress Improves the Composition of Secondary Metabolites in Safflower Flower at the Expense of Reduction in Seed Yield and Oil Content. Ind. Crops Prod..

[59] Baozhu L., Ruonan F., Yanting F., Runan L., Hui Z., Tingting C., Jiong L., Han L., Xiang Z., Chun-peng S. (2022). The Flavonoid Biosynthesis Regulator PFG3 Confers Drought Stress Tolerance in Plants by Promoting Flavonoid Accumulation. Environ. Exp. Bot..

[60] Zhang G., Yu Z., Yao B., Teixeira Da Silva J.A., Wen D. (2022). SsMYB113, a Schima Superba MYB Transcription Factor, Regulates the Accumulation of Flavonoids and Functions in Drought Stress Tolerance by Modulating ROS Generation. Plant Soil.

[61] Huang X., Rong W., Zhang X., Gao Y., Zhou Y., Su J., Luo H., Chu G., Wang M. (2024). Transcriptome and Metabolome Analysis Reveal the Dynamic Changes and Biosynthesis Pathways of Alkaloids in *Sophora alopecuroides* L. under Drought Stress. Ind. Crops Prod..

[62] Guo Z., He S., Zhong X., Yang N., Xu D. (2025). Optimizing Plant Alkaloid Biosynthesis under Drought Stress: Regulatory Mechanisms and Biotechnological Strategies. J. Plant Physiol..

[63] Li X., Zhao Y., Gao C., Li X., Wu K., Lin M., Sun W. (2025). Integrated Analysis of Physiological Responses and Transcriptome of Cotton Seedlings under Drought Stress. Int. J. Mol. Sci..

[64] Claussen W. (2005). Proline as a Measure of Stress in Tomato Plants. Plant Sci..

[65] Liu Q., Li W., Zhao X., Zhang H., Chen J., Liu Q., Li T., Dong S. (2024). Transcriptomic Profiling Analyses Revealed Candidate Genes under Freezing Stress in Siberian Apricot (*Prunus sibirica*). Forests.

[66] Yan W.-J., Pendi F.H., Hussain H. (2022). Improved CTAB Method for RNA Extraction of Thick Waxy Leaf Tissues from Sago Palm (*Metroxylon sagu* Rottb.). Chem. Biol. Technol. Agric..

[67] Liu M.-J., Zhao J., Cai Q.-L., Liu G.-C., Wang J.-R., Zhao Z.-H., Liu P., Dai L., Yan G., Wang W.-J. (2014). The Complex Jujube Genome Provides Insights into Fruit Tree Biology. Nat. Commun..

[68] Love M.I., Huber W., Anders S. (2014). Moderated estimation of fold change and dispersion for RNA-seq data with DESeq2. Genome Biol..

[69] Li S., Deng B., Tian S., Guo M., Liu H., Zhao X. (2021). Metabolic and Transcriptomic Analyses Reveal Different Metabolite Biosynthesis Profiles between Leaf Buds and Mature Leaves in *Ziziphus jujuba* Mill. Food Chem..

[70] Livak K.J., Schmittgen T.D. (2001). Analysis of Relative Gene Expression Data Using Real-Time Quantitative PCR and the 2^−ΔΔCT^ Method. Methods.

